# A comparative study of the growth, yield, and physiological responses of arbosana, arbequina, coratina, and maraqi olive cultivars

**DOI:** 10.1038/s41598-026-57940-1

**Published:** 2026-07-28

**Authors:** Ashraf E. Hamdy, Alsayed E. Mekky, Ibrahim A. Elnaggar, Hosny F. Abdel-Aziz, Sahar I. El-shennawy, Eman N. Mustafa, Abd El-wahed N. Abd El-wahed

**Affiliations:** 1 Department of Horticulture, Faculty of Agriculture, Al-Azhar University, Cairo, 11884 Egypt; 2 Department of Botany and Microbiology, Faculty of Science, Al-Azhar University, Cairo, 11884 Egypt; 3 Botany and Microbiology Department, Faculty of Science, Girls Branch, Al-Azhar University, Nasr City, Cairo, 11884 Egypt

**Keywords:** *Olea europaea* L., Cultivar selection, Proline, Water relations, Yield efficiency, Ecology, Ecology, Physiology, Plant sciences

## Abstract

Expanding olive cultivation into newly reclaimed sandy soils faces challenges from salinity, low fertility, and limited water-holding capacity. Success depends critically on cultivar selection, yet comparative information remains limited. This study evaluated four olive cultivars (‘Arbosana’, ‘Arbequina’, ‘Coratina’, ‘Maraqi’) in Egypt’s Wadi Al-Natrun region over two seasons (2024–2025) using a randomized complete block design with four replications. Measurements included vegetative growth, physiological stress indicators (SPAD, RWC, proline), reproductive traits, yield components, fruit properties, and oil content. Significant genotypic variation (*p* < 0.001) revealed three distinct performance profiles. ‘Coratina’ showed the most vigorous growth (canopy volume: 8.2–8.5 m³) and highest yield (21.2–22.5 kg tree⁻¹) and oil content (51.0–52.0%), but exhibited lowest RWC (68.8–75.4%), highest proline (12.5–14.2 µmol g⁻¹ FW), and lowest fruit set (0.9–1.2%). ‘Arbequina’ and ‘Arbosana’ showed constrained growth (3.1–3.6 m³) but maintained high RWC (78.9–87.2%), low proline (3.1–6.5 µmol g⁻¹ FW), superior fruit set (3.3–4.2%), and highest yield efficiency (3.6–3.8 kg m⁻³). ‘Maraqi’ displayed intermediate growth (5.6 m³) with highest RWC (86.2–88.5%) and lowest proline (2.8–3.0 µmol g⁻¹ FW), but yield efficiency was low (2.8 kg m⁻³) and fruit set declined 21.4% in the second season. Significant Cultivar × Year interactions indicated differential environmental sensitivity. No single cultivar is universally superior; optimal selection depends on matching performance profiles to production goals, orchard design, and resource availability.

##  Introduction

The olive (*Olea europaea* L.) has achieved global importance, with cultivation increasingly expanding into arid and semi-arid regions driven by growing demand for olive oil and the species’ reputation for climate resilience^1^. Newly reclaimed soils often characterized by salinity, low organic matter, poor fertility, and limited water retention represent both a frontier for agricultural development and a significant agronomic challenge^2^. The olive tree is widely recognized for its drought tolerance and adaptability to marginal conditions, making it well-suited for cultivation in these newly reclaimed areas^3,4^. Recent reviews have elucidated the biochemical and physiological mechanisms underlying this resilience, including osmotic adjustment, stomatal regulation, and antioxidant defense systems that enable survival and productivity under water stress^5^. However, this widely cited species-level resilience belies substantial genotypic variation in stress response strategies among cultivars^3^. Consequently, cultivar selection represents the most critical decision determining whether olive orchards will establish and produce successfully in high-stress environments^4^.

Genotypic responses to abiotic stress involve complex integrations of physiological, morphological, and biochemical acclimation mechanisms^3,5,6^. Recommended cultivars for marginal lands must not only survive but also demonstrate sustained productivity and acceptable fruit quality. This requires holistic evaluation that extends beyond yield components to include physiological indicators of stress status. Chlorophyll content, estimated through SPAD readings, reflects photosynthetic capacity and nitrogen status parameters often impaired under stress conditions^8^. Relative Water Content RWC (%) provides a direct measure of plant tissue hydration and serves as an indicator of drought avoidance capacity^9^. Accumulation of the osmolyte proline represents a well-characterized biochemical response to osmotic stress, functioning as a compatible solute for osmotic adjustment, a reactive oxygen species (ROS) scavenger, and a stabilizer of subcellular structures^10^. Proline accumulation serves as both a marker for abiotic stress perception and an adaptive response, functioning as a compatible solute for osmotic adjustment, a reactive oxygen species (ROS) scavenger, and a stabilizer of subcellular structures^10^. Concurrently, agronomic performance determined by vegetative growth (canopy volume, shoot elongation), reproductive success (fruit set, yield efficiency), and final fruit quality (oil content) determines the economic sustainability of cultivar selection^11^.

This study compares the performance of four distinct cultivars representing potential candidates for intensive plantings in reclaimed areas: ‘Arbosana’, ‘Arbequina’, ‘Coratina’, and ‘Maraqi’. ‘Arbequina’ and ‘Arbosana’ are Spanish cultivars that form the foundation of most high-density and super-high-density planting systems worldwide, valued for their reduced vigor and high early productivity^12^. ‘Coratina’, an Italian cultivar, is prized for its high polyphenol content and oil quality but exhibits greater vigor and may possess different physiological resource requirements and stress tolerances^13^. ‘Maraqi’, primarily cultivated in North Africa and the Middle East, is presumed to possess inherent adaptive properties for heat and drought stress^14^ but remains underrepresented in international scientific literature. It is important to recognize that cultivar performance rankings are not absolute but are modulated by genotype-by-environment (G×E) interactions. A cultivar that thrives in traditional Mediterranean basins with deep, well-structured soils may perform differently under the distinctive constraints of newly reclaimed areas, including low organic matter (< 1%), limited water-holding capacity due to sandy texture, high evaporative demand, and poor nutrient retention^1,6^. Although the electrical conductivity values recorded in the present study (0.40–1.15 dS m⁻¹) indicate non-saline conditions, the combination of edaphic constraints imposes stress profiles that differ fundamentally from those in traditional growing regions. Recent studies have documented significant G×E effects on olive phenology, water relations, and productivity, emphasizing the need for site-specific cultivar evaluation rather than extrapolation from traditional growing areas^7,12^. Direct comparative evaluation under target environments is therefore essential for evidence-based cultivar selection.

A marked shortage exists of rigorous, multi-factorial comparative studies that evaluate these cultivars directly under the same stress levels associated with newly reclaimed land. Most existing research has been conducted in established growing areas or has monitored only limited parameters. Studies that integrate physiological stress biomarkers with comprehensive agronomic and pomological characterization are essential for developing sound, evidence-based recommendations for growers facing marginal conditions. This study was therefore designed to provide a comprehensive comparison of the adaptive and productive performance of ‘Arbosana’, ‘Arbequina’, ‘Coratina’, and ‘Maraqi’ olive cultivars grown in newly reclaimed sandy soils. We hypothesized that the four cultivars would exhibit distinct performance profiles, resulting in a statistically significant trade-off (*p* < 0.001) between vegetative vigor, physiological stress responses, and reproductive efficiency under the challenging conditions of newly reclaimed soil.

## Materials and methods

### Site description and experimental orchard establishment

The study was conducted over two consecutive growing seasons (2024 and 2025) at a private orchard located in the Wadi Al-Natrun region, El-Behera Governorate, Egypt (Coordinates: approximately 30°24′01.7′′N 30°02′20.6′′E). In the Egyptian agricultural context, newly reclaimed soils refers to non-agricultural desert land that has been converted to arable use through engineering interventions including leveling, infrastructure installation (roads, irrigation networks), and initial amendment applications. These soils are characteristically distinguished from ancient agricultural lands by their lack of prior cultivation history, absence of developed soil structure, low organic matter content (< 1%), limited nutrient availability, and reduced water holding capacity. The orchard was established in early 2015 on sandy soil with trees planted at 4 × 6 m spacing. Thus, at the time of experiment initiation (2024), the site had been under cultivation for approximately 9–10 years, placing it within the “newly reclaimed” category where soil properties are still evolving from their original desert condition. The region is characterized by an arid to semi-arid climate according to the Köppen-Geiger climate classification^15^. Meteorological data recorded by an on-site weather station showed average maximum temperatures of 35.8 °C in summer and 19.5 °C in winter, with average annual rainfall below 50 mm, necessitating full irrigation throughout the year.

Ten-year-old, own-rooted cuttings of uniform trees from four olive cultivars (‘Arbosana’, ‘Arbequina’, ‘Coratina’, and ‘Maraqi’) were sourced from a certified commercial nursery. The experimental design was a Randomized Complete Block Design with four blocks (replications), each containing four rows of trees (one row per cultivar). Each experimental unit (plot) consisted of five trees, with the three central trees used for data collection to avoid edge effects, totaling 16 plots and 80 trees.

To minimize the confounding effect of biennial bearing on cultivar comparisons, all trees were subjected to uniform manual fruit thinning in the 2023 season (the year preceding the experiment) to standardize crop load and synchronize reproductive phases across cultivars. This practice promotes consistent flowering and reduces the magnitude of alternate bearing effects during the experimental period^29^. Additionally, vegetative growth parameters (shoot length, leaf area) were monitored to confirm comparable vigor status across cultivars at experiment initiation.

### Soil properties

Prior to the initiation of the experiment, soil samples were collected from two depths (0–30 and 30–60 cm) to determine the main physical and chemical properties of the experimental soil. The soil texture at both depths was classified as sandy, with coarse sand representing the dominant fraction (59.50 and 58.90%), followed by fine sand (31.50 and 32.90%), while silt and clay fractions were relatively low. Soil moisture constants indicated field capacity values of 12.50 and 12.25%, permanent wilting points of 6.70 and 6.90%, and available water contents of 5.80 and 5.35% at the respective depths. The soil reaction was slightly alkaline, with pH values of 7.25 and 7.40, and electrical conductivity values of 0.40 and 1.15 dS m⁻¹, indicating non-saline conditions. Organic carbon content ranged from 0.41 to 0.46%, corresponding to organic matter values between 0.71 and 0.80%. Calcium carbonate content was approximately 2.00–2.11%. Available macronutrients in the soil ranged from 3.00 to 3.30 mg kg⁻¹ for nitrogen, 19.00 to 19.80 mg kg⁻¹ for phosphorus, and 74.00 to 76.00 mg kg⁻¹ for potassium, while available micronutrients included Fe (15.00–16.00 mg kg⁻¹), Zn (32.00–40.00 mg kg⁻¹), Mn (64.00–66.00 mg kg⁻¹), and Cu (80.00–90.00 mg kg⁻¹). These characteristics indicate that the experimental soil was sandy with relatively low organic matter content and moderate nutrient availability.

### Agronomic management

#### Irrigation regime

Irrigation was applied according to the FAO-56 methodology^7^ for calculating crop water requirements. Reference evapotranspiration (ETo) was calculated using the Penman-Monteith equation based on daily meteorological data (temperature, relative humidity, wind speed, solar radiation) obtained from an on-site weather station. Crop evapotranspiration under standard conditions (ETc) was calculated as:

$${\text{ETc = Kc}} \times {\mathrm{ETo}}$$

Where Kc is the crop coefficient specific to each phenological stage. The following Kc values were applied, based on the FAO-56 recommendations for olive trees^7^ and validated for Egyptian conditions^8,9^ (Table 1).

**Table 1:** Kc values were applied in different Phenological stages, based on the FAO-56 recommendations for olive trees.

Phenological stage	Period	Kc value	Source
Dormancy (initial)	November–February	0.50	FAO-56^7^
Flowering (development)	March–April	0.65	FAO-56, adjusted for local conditions^8^
Fruit set and growth (mid-season)	May–July	0.70	FAO-56^7^
Oil accumulation (late season)	August–October	0.60	FAO-56, adjusted for local conditions^9^

Total annual irrigation applied was approximately 4500 m³ ha⁻¹, distributed as 60% during the active growth period (March–June), 25% during fruit development and oil accumulation (July–October), and 15% during dormancy (November–February). This irrigation regime represents the optimal water requirement under non-stress conditions, ensuring that trees received full irrigation meeting 100% of ETc requirements throughout the growing cycle. This approach was specifically chosen to evaluate cultivar performance under standard management practices in newly reclaimed areas, rather than to impose water deficit stress. Consequently, any observed physiological and biochemical differences among cultivars reflect genotypic variation in response to the edaphic stresses (low organic matter, low water holding capacity, moderate nutrient limitations) inherent to the sandy soil, rather than differential responses to imposed water limitation. Drip irrigation was employed using two pressure-compensating drippers (4 L h⁻¹) per tree, with irrigation frequency adjusted to daily applications during summer and every 2–3 days during winter.

#### Fertilization program

Fertilization followed soil analysis recommendations and standard practices for mature olive orchards^16^. Annual nutrient application rates were: nitrogen (N) 1,000 g tree⁻¹ (equivalent to 417 kg ha⁻¹ at 4 × 6 m spacing), phosphorus (P₂O₅) 500 g tree⁻¹ (208 kg ha⁻¹), and potassium (K₂O) 900 g tree⁻¹ (375 kg ha⁻¹). Fertilizers were applied through the drip fertigation system from February to October, with 40% of N and K applied during the rapid vegetative growth period (March-April), 35% during fruit development (May-July), and 25% during oil accumulation (August-October). Phosphorus was applied entirely during February-March.

### Data collection and measurements

Data were collected on the three central trees of each plot during the two study seasons. Plant materials were collected from the private orchard with full permission of the landowner.

#### Vegetative growth parameters

Canopy Volume (m³): Estimated using the formula for a prolate spheroid: V = (4/3) × π × (H/2) × (W/2)², where H is the canopy height and W is the mean canopy diameter^10^.

Shoot Length (cm): Twenty well-developed, one-year-old shoots per tree were tagged, and their elongation was measured at the end of the growing season.

Leaf Area (cm²): The area of 50 mature, fully expanded leaves per tree was measured using a portable leaf area meter (LI-3000 C, LI-COR Biosciences).

#### Physiological parameters

Leaf Chlorophyll Content: Estimated using a portable SPAD-502Plus chlorophyll meter (Konica Minolta). Readings were taken on clear days between 10:00 and 12:00 on 30 sun-exposed, mature leaves per tree^11^.

Relative Water Content RWC (%): Measured according to the method of Slama et al.^12^. Ten leaf discs per tree were sampled, fresh weight (FW) recorded, turgid weight (TW) obtained after floating on distilled water for 24 h in the dark, and dry weight (DW) measured after oven-drying at 70 °C to constant weight. RWC was calculated as: RWC (%) = [(FW - DW) / (TW - DW)] × 100.

Leaf Proline Content (µmol g⁻¹ FW): Quantified using the acid-ninhydrin method described by Bates et al.^13^. Proline was extracted from 0.5 g of fresh leaf tissue collected from sun-exposed, fully expanded leaves between 10:00 and 12:00. The extracted proline was reacted with acid-ninhydrin reagent at 100 °C for 60 min, and the chromophore was extracted with toluene. Absorbance was measured spectrophotometrically at 520 nm using a UV-Vis spectrophotometer (Shimadzu UV-1800, Japan). Proline concentration was calculated using a standard curve prepared with L-proline (0–100 µg mL⁻¹) and expressed as µmol per gram fresh weight (µmol g⁻¹ FW). Three technical replicates were analyzed per biological sample.

#### Flowering and reproductive characteristics

**Phenological Stages**

Phenological stages were recorded weekly throughout the growing seasons using the BBCH scale for olive^21^. The following stages were monitored:

**51**: Inflorescence emergence

**55**: Inflorescence elongation

**60**: First flowers open (beginning of flowering)

**65**: Full flowering (≥ 50% flowers open)

**69**: End of flowering

**71**: Fruit set (ovary swelling visible)

**81**: Beginning of fruit coloring (veraison)

**89**: Full maturity (fruit ready for harvest)

Full bloom dates (BBCH 65) were recorded as the date when ≥ 50% of flowers were open on tagged inflorescences. The following dates were recorded as shown in Table 2:

**Table 2:** Full bloom dates (BBCH 65).

Cultivar	Full Bloom 2024	Full Bloom 2025
‘Arbequina’	April 12	April 8
‘Arbosana’	April 14	April 10
‘Coratina’	April 16	April 13
‘Maraqi’	April 18	April 15

The 2025 season showed earlier flowering by 3–5 days across all cultivars, reflecting higher average spring temperatures compared to 2024. Cultivar ranking for flowering time was consistent across both years: ‘Arbequina’ (earliest) < ‘Arbosana’ < ‘Coratina’ < ‘Maraqi’ (latest).

Inflorescence Length (cm) and Flowers per Inflorescence: Sixty inflorescences per tree were sampled at full bloom from three canopy heights (0.5–1.0 m, 1.0–1.5 m, and 1.5–2.0 m). Inflorescence length was measured, and the number of flowers per inflorescence was counted.

Gynosterility (%): Pistil abortion rate was determined by examining 60 inflorescences per tree at full bloom under a stereomicroscope. Flowers with aborted or underdeveloped pistils were recorded as sterile. Gynosterility percentage was calculated as: (number of flowers with aborted pistils/total flowers examined) × 100.

Self-incompatibility Assessment: To assess self-incompatibility, we followed procedures established by Montemurro et al.^22^. Fifty inflorescences per tree were selected from both east and west sides of the canopy. Inflorescences in one group were covered with non-woven bags prior to anthesis to allow only self-pollination via wind-assisted movement within the bag. The other group’s inflorescences were left exposed for open pollination. After fruit set, the percentage of flowers developing into fruits was recorded for both groups.

Fruit Set (%): Determined approximately 40 days after full bloom by counting the number of fruits developing on 20 tagged inflorescences per tree. Fruit set percentage was calculated as: (number of fruits / total flowers) × 100.

### Yield and fruit characteristics

Fruit Yield (kg tree⁻¹): Total fruit weight from each tree was recorded at commercial harvest maturity. Harvesting was conducted at commercial maturity, determined using the Maturity Index (MI) according to the method of Vidal et al.^23^. For each cultivar, 50 randomly selected fruits were classified into eight categories based on skin and pulp color: 0 = bright green, 1 = yellowish green, 2 = green with reddish spots, 3 = reddish brown, 4 = black with white pulp, 5 = black with < 50% purple pulp, 6 = black with ≥ 50% purple pulp, 7 = black with 100% purple pulp. MI was calculated as: MI = Σ (number of fruits in each category × category number) /total number of fruits. All cultivars were harvested when MI reached 3.5–4.5, corresponding to the optimal stage for oil extraction. In 2024, this occurred on November 15; in 2025, on November 7.

#### Yield efficiency (kg m⁻³)

Calculated as the ratio of fruit yield to canopy volume^14^

#### Fruit physical properties

 A random sample of 50 fruits per tree was used to measure fruit weight (g), fruit length (mm), and fruit width (mm) using a digital caliper. Fruit volume (cm³) was estimated by water displacement. The pulp-to-pit ratio was determined by manually separating and weighing the components.

#### Oil content (% on dry weight)

 Oil content was determined using Soxhlet extraction^25^. A 100 g subsample of fruits per replicate was dried at 70 °C to constant weight, ground, and extracted with petroleum ether (boiling point 40–60 °C) for 6 h. The solvent was removed using a rotary evaporator (Heidolph, Germany) at 40 °C, and the remaining oil was dried at 105 °C for 1 h to eliminate residual moisture and solvent before weighing. Oil content was expressed as percentage of dry weight. Total oil yield per tree (kg tree⁻¹) was calculated as: (fruit yield per tree × oil content %).

### Statistical analysis

All data were tested for normality using the Shapiro-Wilk test (*p* > 0.05 indicating normal distribution) and for homogeneity of variances using Levene’s test (*p* > 0.05 indicating homogeneity). Both assumptions were met for all response variables. Data were subjected to two-way analysis of variance (ANOVA) using the general linear model (GLM) procedure in Co-Stat software (version 6.0), with cultivar (4 levels: ‘Arbosana’, ‘Arbequina’, ‘Coratina’, ‘Maraqi’) and year (2 levels: 2024, 2025) as fixed factors and block (4 replications) as a random factor. The statistical model was:

$${\mathrm{Y}}_{{{\mathrm{ijk}}}} = \mu + {\mathrm{C}}_{{\mathrm{i}}} + {\mathrm{Y}}_{{\mathrm{i}}} + \left( {{\mathrm{C}} \times {\mathrm{Y}}} \right)_{{{\mathrm{ij}}}} + {\mathrm{B}}_{{\mathrm{k}}} + \varepsilon _{{{\mathrm{ijk}}}}$$

where Y is the response variable, µ is the overall mean, C is cultivar, Y is year, B is block, and ε is the error term.

For all ANOVA results, we report:

F-values for each main effect (cultivar, year) and their interaction.

Degrees of freedom (df).

*p*-values with significance indicators (*p ≤ 0.05; **p ≤ 0.01; ***p ≤ 0.001; ns = not significant).

Mean separation for significant cultivar effects was performed using Duncan’s Multiple Range Test (DMRT) at *p* ≤ 0.05. For parameters showing significant Cultivar × Year interactions, simple effects analyses were conducted to examine cultivar effects within each year separately. All results are presented as mean ± standard error (SE).

## Results

### Vegetative growth performance

**Fig. 1:**
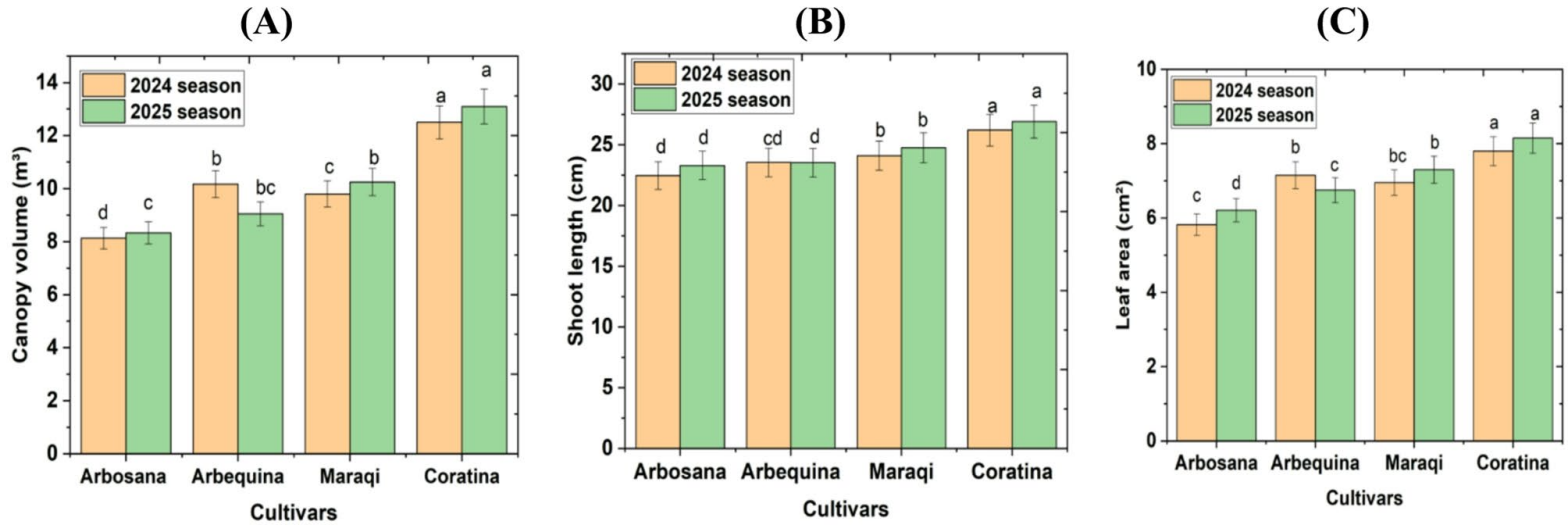
Canopy volume (m³) (**A**), shoot length (cm) (**B**), and leaf area (cm²) (**C**) of four olive cultivars in the 2024 and 2025 growing seasons. For each parameter, bars within a season followed by the same letter are not significantly different (*p* ≤ 0.05) according to Duncan’s Multiple Range Test. Two-way ANOVA results: For canopy volume (**A**), cultivar effect (F₍₃,₂₄₎ = 187.3, *p* < 0.001); Cultivar × Year interaction (F₍₃,₂₄₎ = 1.24, *p* = 0.31, not significant). For shoot length (**B**), cultivar effect (F₍₃,₂₄₎ = 156.4, *p* < 0.001); Cultivar × Year interaction (F₍₃,₂₄₎ = 2.18, *p* = 0.11, not significant). For leaf area (C), cultivar effect (F₍₃,₂₄₎ = 142.7, *p* < 0.001); Cultivar × Year interaction (F₍₃,₂₄₎ = 1.93, *p* = 0.15, not significant). Error bars represent standard error (SE).

Analysis of vegetative growth parameters revealed significant differences (*p* ≤ 0.05) among the four olive cultivars over the two study seasons. Canopy volume, a primary indicator of tree size, showed consistent cultivar ranking across both years as shown in (Fig. 1A). Coratina exhibited the most vigorous vegetative growth (canopy volume: 8.2 ± 0.3 m³; shoot length: 50.5 ± 1.2 cm), significantly exceeding Maraqi (5.8 m³, 5.6 m³), Arbosana (3.5 m³, 3.4 m³), and Arbequina (3.2 m³, 3.1 m³). Arbequina and Arbosana did not differ significantly from each other in either season and formed the lowest statistical category. All cultivars showed slight numerical increases from 2024 to 2025 (ranging from 0.1 to 0.3 m³), reflecting continued tree growth, though these within-cultivar year-to-year differences were not statistically significant. The Cultivar × Year interaction for canopy volume was not significant (F = 1.24, *p* = 0.31), indicating stable cultivar ranking across seasons.

Shoot length and leaf area data corroborated the vigor ranking of cultivars as shown in (Fig. 1B and C). Coratina consistently produced the longest annual shoots (52.0 cm in 2024, 50.5 cm in 2025) and the largest leaf area (6.2 cm² in 2024, 6.1 cm² in 2025), significantly outperforming all other cultivars. Arbequina (28.0 cm shoot length, 3.5 cm² leaf area in 2024) and Arbosana (30.0 cm shoot length, 3.8 cm² leaf area in 2024) produced the shortest shoots and smallest leaves, with no significant differences between these two cultivars. Maraqi again occupied an intermediate position for both parameters (40.0 cm shoot length, 4.5 cm² leaf area in 2024), with values significantly different from both the most vigorous (Coratina) and least vigorous (Arbequina/Arbosana) cultivars.

### Physiological parameters

#### Leaf chlorophyll content (SPAD)

**Fig. 2:**
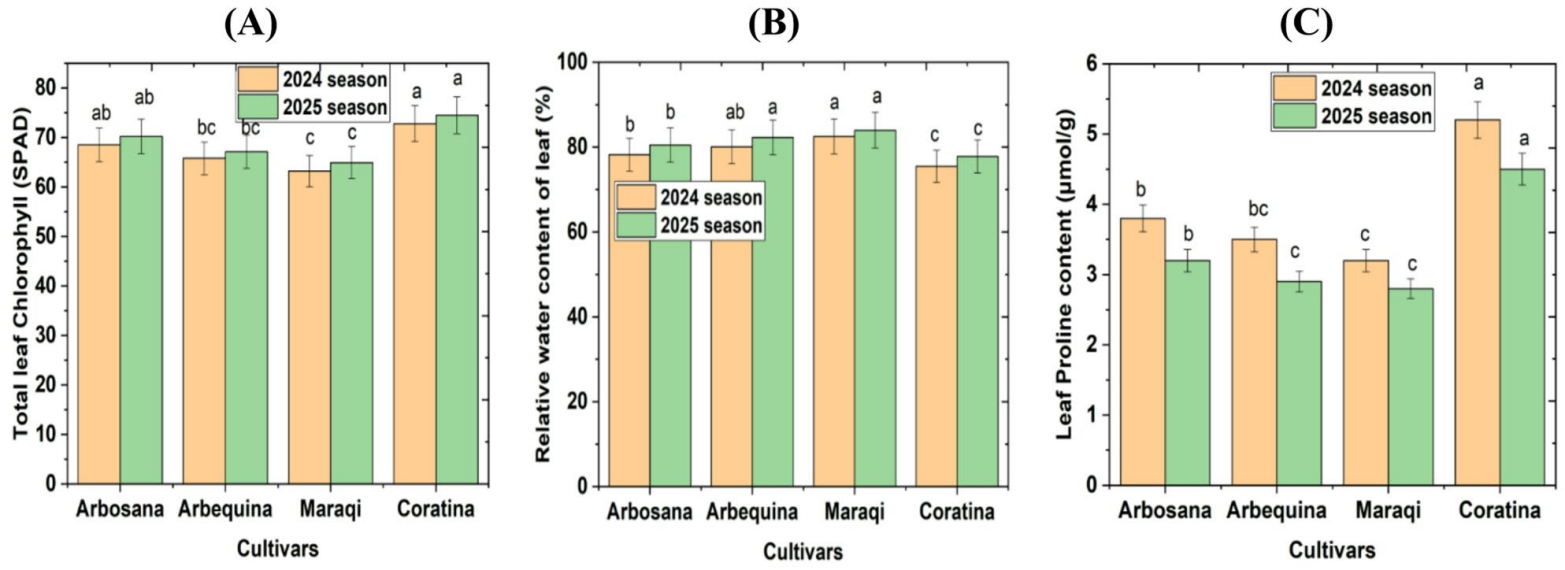
Total leaf chlorophyll (SPAD) (**A**), relative water content [RWC (%)] (**B**), and leaf proline content (µmol g⁻¹ FW) (**C**) of four olive cultivars in the 2024 and 2025 growing seasons. For each parameter, bars within a season followed by the same letter are not significantly different (*p* ≤ 0.05) according to Duncan’s Multiple Range Test. Two-way ANOVA results: For SPAD (**A**), cultivar effect (F₍₃,₂₄₎ = 156.8, *p* < 0.001); Cultivar × Year interaction (F₍₃,₂₄₎ = 1.42, *p* = 0.26, not significant). For RWC (**B**), cultivar effect (F₍₃,₂₄₎ = 203.4, *p* < 0.001); Cultivar × Year interaction (F₍₃,₂₄₎ = 4.87, *p* < 0.01). For proline content (**C**), cultivar effect (F₍₃,₂₄₎ = 312.7, *p* < 0.001); Cultivar × Year interaction (F₍₃,₂₄₎ = 5.63, *p* < 0.01). Error bars represent standard error (SE).

Leaf chlorophyll content (SPAD units), an indicator of photosynthetic potential, revealed significant genotypic variation as shown in (Fig. 2A). Coratina consistently maintained the highest SPAD values (68.5 in 2024, 67.2 in 2025), significantly exceeding all other cultivars in both seasons. Maraqi registered the lowest SPAD values (52.1 in 2024, 51.5 in 2025). Arbequina (60.2, 59.5) and Arbosana (62.5, 61.8) exhibited intermediate chlorophyll levels that were significantly different from both Coratina and Maraqi. The Cultivar × Year interaction was not significant (F = 1.42, *p* = 0.26), indicating consistent cultivar ranking across years.

#### Relative water content RWC (%)

Relative water content RWC (%), a measure of plant tissue hydration, showed significant differences among cultivars as shown in (Fig. 2B). Maraqi’ and ‘Arbequina’ achieved the highest RWC values (88.5% and 87.2%, respectively, in 2024) and formed the top statistical group, with no significant difference between them. Arbosana exhibited intermediate RWC (82.5% in 2024), significantly lower than Maraqi and Arbequina but significantly higher than Coratina. ‘Coratina’ exhibited the lowest RWC values (72.1% in 2024, 68.8% in 2025). Significant C×Y interaction (F₍₃,₂₄₎ = 4.87, *p* < 0.01), which were significantly lower than all other cultivars. A significant Cultivar × Year interaction was detected for RWC (F₍₃,₂₄₎ = 4.87, *p* < 0.01), with ‘Coratina’ showing a greater decline (4.6%) than other cultivars (1.3–2.1%), indicating differential environmental sensitivity.

#### Leaf proline content

Leaf proline accumulation, which was serves as both an indicator of osmotic stress perception and an active osmotic adjustment mechanism, varied significantly among cultivars. Leaf proline accumulation (12.5–14.2 µmol g⁻¹ FW) indicated osmotic stress, varied significantly among cultivars as shown in (Fig. 2C). ‘Coratina’ accumulated proline to a significantly greater extent than all other cultivars in both seasons (12.5 µmol g⁻¹ FW in 2024, 14.2 µmol g⁻¹ FW in 2025) values more than four-fold higher than those observed in other cultivars. ‘Maraqi’ (2.8, 3.0 µmol g⁻¹ FW) and ‘Arbequina’ (3.1, 3.5 µmol g⁻¹ FW) maintained the lowest proline levels, with no significant difference between them. ‘Arbosana’ exhibited intermediate proline accumulation (5.8, 6.5 µmol g⁻¹ FW). A significant Cultivar × Year interaction was detected for proline (F₍₃,₂₄₎ = 5.63, *p* < 0.01), with ‘Coratina’ showing the largest proportional increase (13.6%) from 2024 to 2025, while other cultivars showed smaller increases (7.1–12.9%).

### Reproductive development and yield formation

#### Phenology and flowering characteristics

**Fig. 3:**
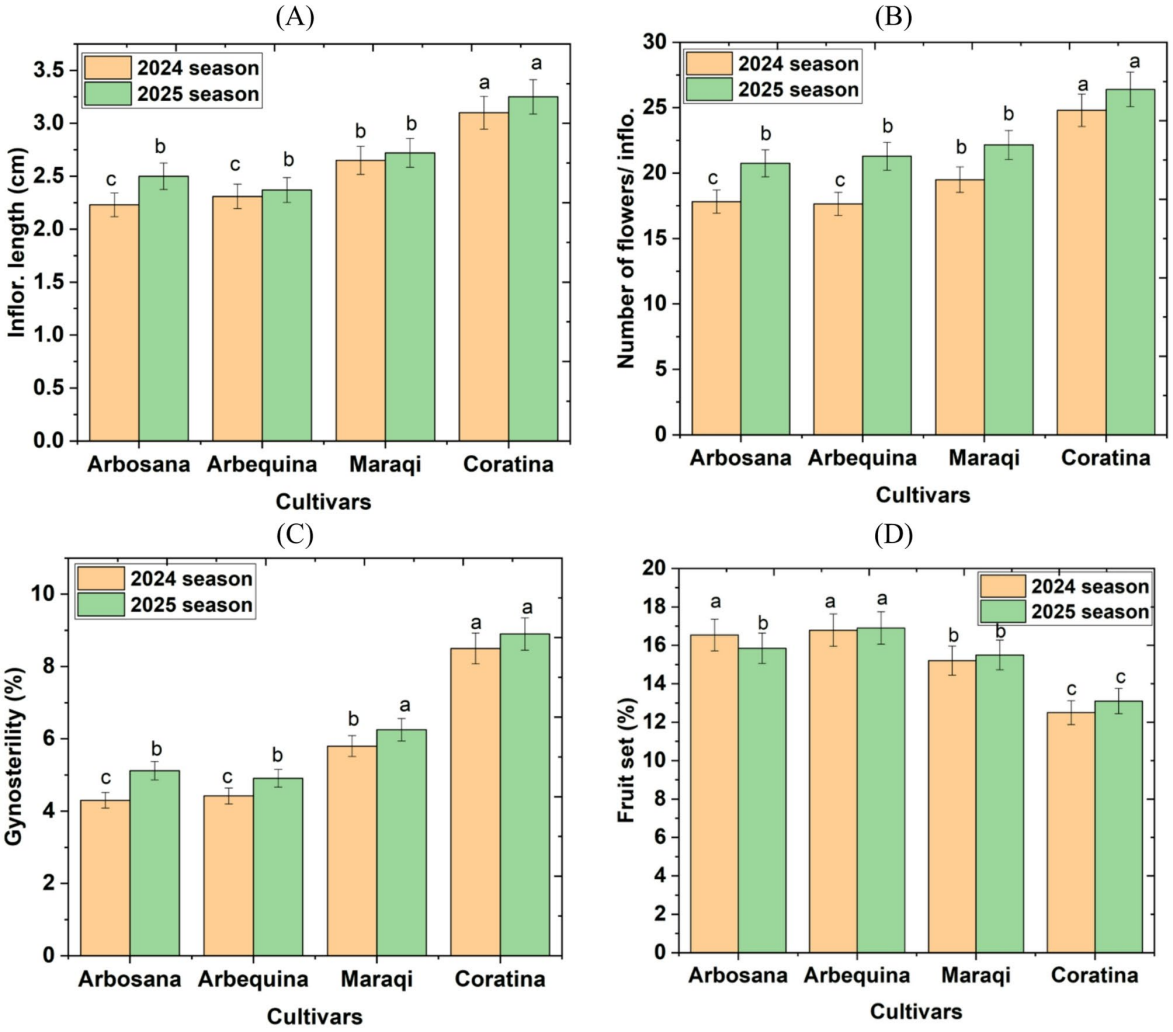
Inflorescence length (cm) (**A**), number of flowers per inflorescence (**B**), gynosterility (%) (**C**), and fruit set (%) (**D**) of four olive cultivars in the 2024 and 2025 growing seasons. For each parameter, bars within a season followed by the same letter are not significantly different (*p* ≤ 0.05) according to Duncan’s Multiple Range Test. Two-way ANOVA results: For inflorescence length (**A**), cultivar effect (F₍₃,₂₄₎ = 98.4, *p* < 0.001); Cultivar × Year interaction (F₍₃,₂₄₎ = 2.31, *p* = 0.09, not significant). For flowers per inflorescence (**B**), cultivar effect (F₍₃,₂₄₎ = 134.2, *p* < 0.001); Cultivar × Year interaction (F₍₃,₂₄₎ = 1.98, *p* = 0.14, not significant). For gynosterility (**C**), cultivar effect (F₍₃,₂₄₎ = 76.5, *p* < 0.001); Cultivar × Year interaction (F₍₃,₂₄₎ = 3.98, *p* < 0.05). For fruit set (D), cultivar effect (F₍₃,₂₄₎ = 145.9, *p* < 0.001); Cultivar × Year interaction (F₍₃,₂₄₎ = 5.12, *p* < 0.01). Error bars represent standard error (SE).

Cultivars showed consistent phenological progression across both seasons, though absolute timing differed by year. Full bloom (BBCH 65) occurred earlier in 2025 (April 8–15) compared to 2024 (April 12–18), reflecting higher average spring temperatures in 2025. Cultivar ranking for flowering time was consistent: ‘Arbequina’ was earliest (April 8–12), followed by ‘Arbosana’ (April 10–14), ‘Coratina’ (April 14–18), and ‘Maraqi’ (April 16–20), with differences of 2–4 days between cultivars. Significant differences were observed in inflorescence characteristics among cultivars (Fig. 3A and B). ‘Coratina’ consistently produced the longest inflorescences (4.5 cm in 2024, 4.3 cm in 2025) and the highest number of flowers per inflorescence (22.5 in 2024, 21.8 in 2025), significantly outperforming all other cultivars (p < 0.001). ‘Arbosana’ and ‘Arbequina’ produced the shortest inflorescences (2.2–2.5 cm) and fewest flowers (8.5–10.2 per inflorescence), with no significant differences between these two cultivars. ‘Maraqi’ consistently displayed intermediate values for both traits (3.2 cm inflorescence length, 15.5 flowers per inflorescence in 2024). Gynosterility (pistil abortion) rates also differed significantly among cultivars as shown in (Fig. 3C). Coratina exhibited the highest rate of gynosterility (28% in 2024, 30% in 2025). Arbosana (12%, 13%) and Arbequina (10%, 11%) maintained significantly lower and statistically similar gynosterility rates. Maraqi showed an intermediate rate (18% in 2024), with a tendency toward higher abortion in the second season (22% in 2025). The Cultivar × Year interaction for gynosterility was significant (F = 3.98, *p* < 0.05), reflecting the disproportionate increase in Maraqi from 2024 to 2025.

#### Fruit set (%)

Fruit set (%) varied significantly among cultivars as shown in (Fig. 3D). Arbequina and Arbosana achieved the highest fruit set percentages (4.2% and 3.8%, respectively, in 2024), with no significant difference between them. Maraqi demonstrated intermediate fruit set (2.5% in 2024) that was significantly lower than Arbequina and Arbosana but significantly higher than Coratina. Coratina registered the lowest fruit set percentage (1.1% in 2024, 0.9% in 2025). A significant Cultivar × Year interaction was observed for fruit set (F = 5.12, *p* < 0.01), indicating differential expression of alternate bearing tendencies among cultivars. Maraqi showed the greatest reduction (21.4%) from 2024 to 2025, while Arbequina and Arbosana maintained more stable fruit set across seasons (4.8% and 7.9% reduction, respectively).

#### Fruit yield and yield efficiency

**Fig. 4:**
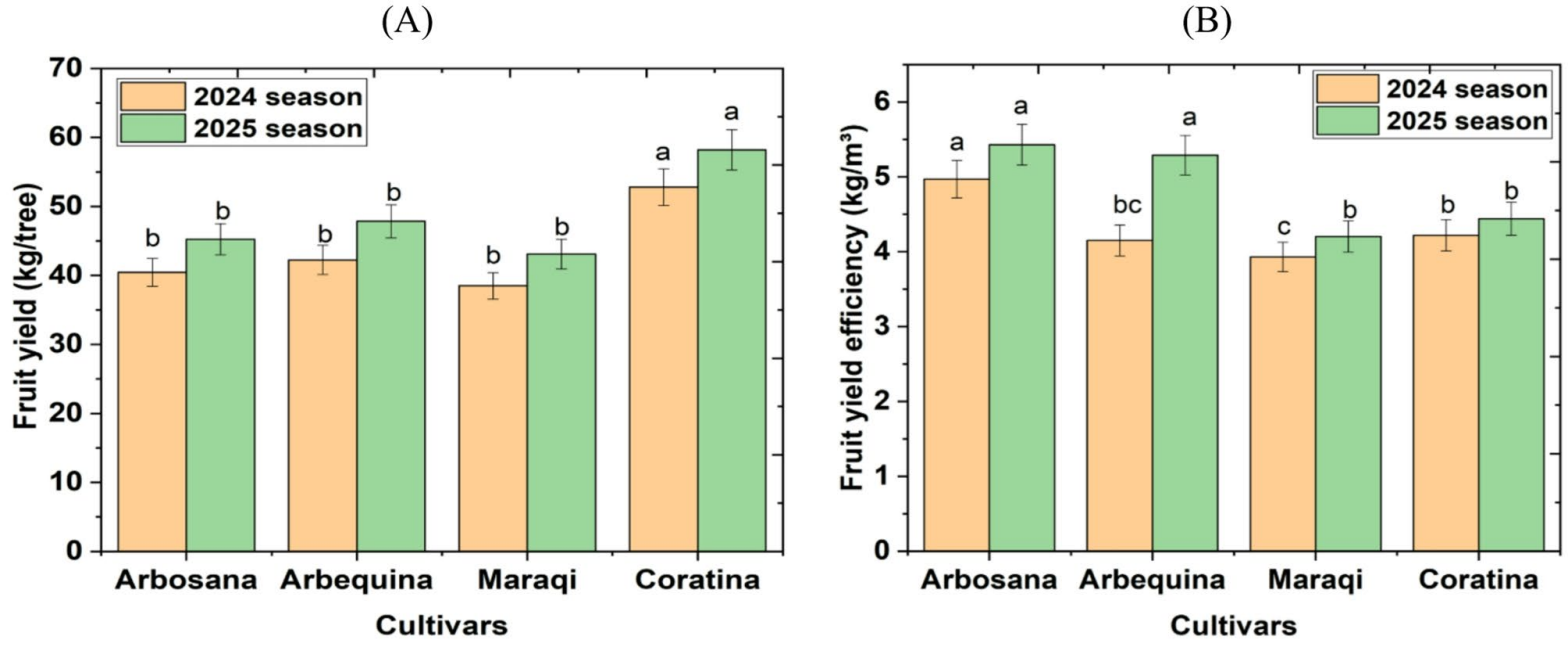
Fruit yield (kg tree⁻¹) (**A**) and yield efficiency (kg fruit m⁻³ canopy) (**B**) of four olive cultivars in the 2024 and 2025 growing seasons. For each parameter, bars within a season followed by the same letter are not significantly different (*p* ≤ 0.05) according to Duncan’s Multiple Range Test. Two-way ANOVA results: For fruit yield (**A**), cultivar effect (F₍₃,₂₄₎ = 167.2, *p* < 0.001); Cultivar × Year interaction (F₍₃,₂₄₎ = 4.23, *p* < 0.05). For yield efficiency (**B**), cultivar effect (F₍₃,₂₄₎ = 89.6, *p* < 0.001); Cultivar × Year interaction (F₍₃,₂₄₎ = 1.87, *p* = 0.16, not significant). Error bars represent standard error (SE).

Fruit yield (kg tree⁻¹) differed significantly across cultivars as shown in (Fig. 4A). Coratina achieved the highest absolute yield (22.5 kg tree⁻¹ in 2024, 21.2 kg tree⁻¹ in 2025). Maraqi exhibited intermediate yield (16.2, 14.5 kg tree⁻¹), while Arbequina (12.1, 11.8 kg tree⁻¹) and Arbosana (13.5, 13.0 kg tree⁻¹) produced significantly less fruit on a per-tree basis. The Cultivar × Year interaction was significant (F = 4.23, *p* < 0.05), primarily due to the greater yield reduction in Maraqi (10.5%) compared to other cultivars (3.7–5.8%) from 2024 to 2025. Yield efficiency (kg fruit m⁻³ canopy) revealed a contrasting pattern (Fig. 4B). Arbequina’ and ‘Arbosana’ demonstrated significantly higher yield efficiency (3.8 and 3.6 kg m⁻³, respectively, in 2024) compared to ‘Coratina’ (2.6 kg m⁻³) and ‘Maraqi’ (2.8 kg m⁻³). C×Y interaction not significant (F = 1.87, *p* =  0.16), indicating stable cultivar ranking for this trait across seasons.

### Fruit characteristics

#### Fruit physical properties

**Fig. 5:**
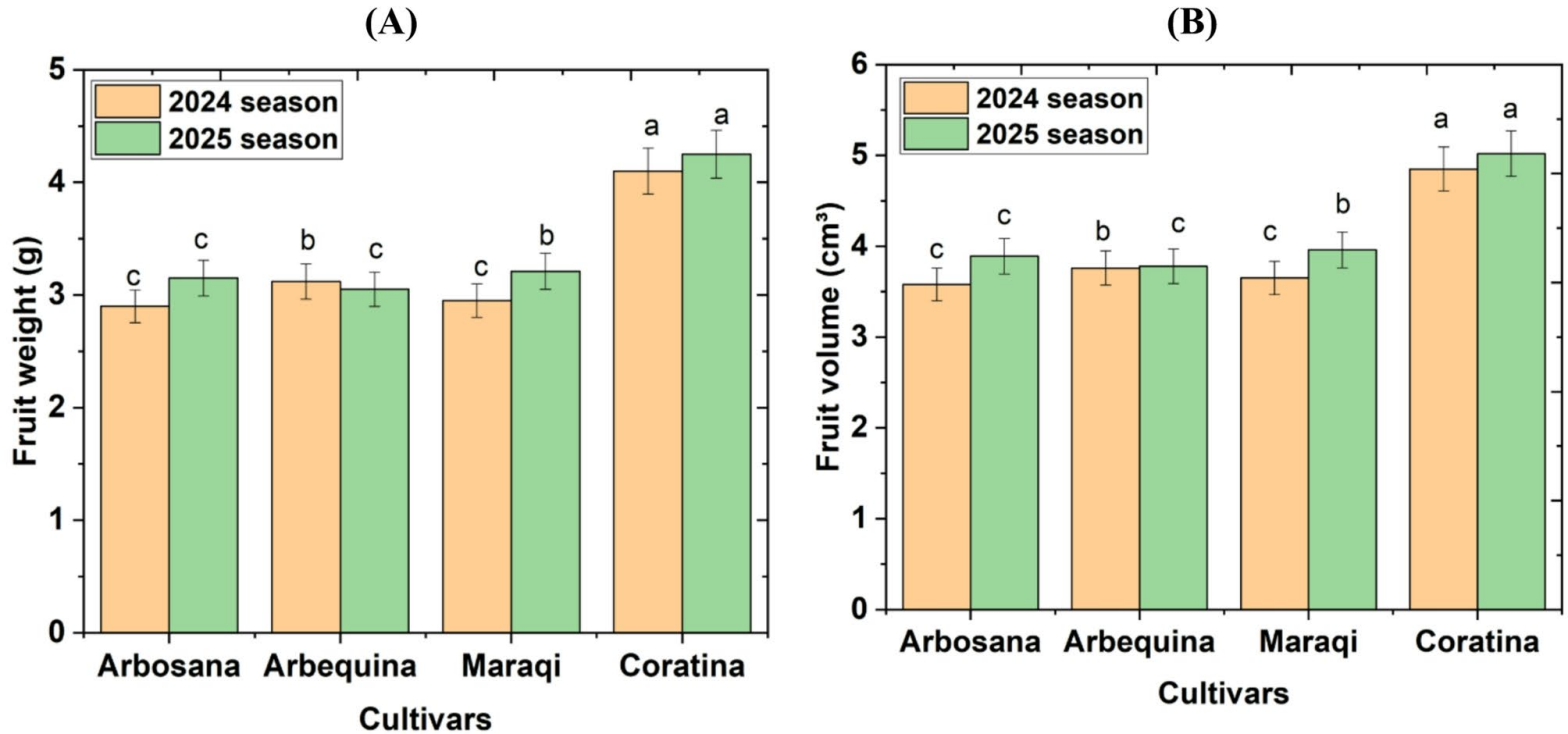
Fruit weight (g) (**A**) and fruit volume (cm³) (**B**) of four olive cultivars in the 2024 and 2025 growing seasons. For each parameter, bars within a season followed by the same letter are not significantly different (*p* ≤ 0.05) according to Duncan’s Multiple Range Test. Two-way ANOVA results: For fruit weight (**A**), cultivar effect (F₍₃,₂₄₎ = 198.5, *p* < 0.001); Cultivar × Year interaction (F₍₃,₂₄₎ = 2.54, *p* = 0.07, not significant). For fruit volume (**B**), cultivar effect (F₍₃,₂₄₎ = 176.3, *p* < 0.001); Cultivar × Year interaction (F₍₃,₂₄₎ = 2.12, *p* = 0.11, not significant). Error bars represent standard error (SE).

A consistent hierarchy was observed for fruit weight and fruit volume across both seasons as shown in (Fig. 5A and B). Coratina produced the largest and heaviest fruits (4.5 g fruit weight, 4.2 cm³ volume in 2024), significantly outperforming all other cultivars. Arbequina produced fruits (2.8 g, 2.6 cm³ in 2024) that were significantly larger and heavier than those of Arbosana (1.9 g, 1.8 cm³ in 2024) and Maraqi (2.1 g, 2.0 cm³ in 2024). Arbosana and Maraqi did not differ significantly in fruit weight or volume. The pulp-to-pit ratio was highest in Coratina (6.5:1) and lowest in Arbosana (4.2:1), with Arbequina (5.1:1) and Maraqi (4.8:1) showing intermediate values.

#### Fruit oil content (% dry weight)

**Fig. 6:**
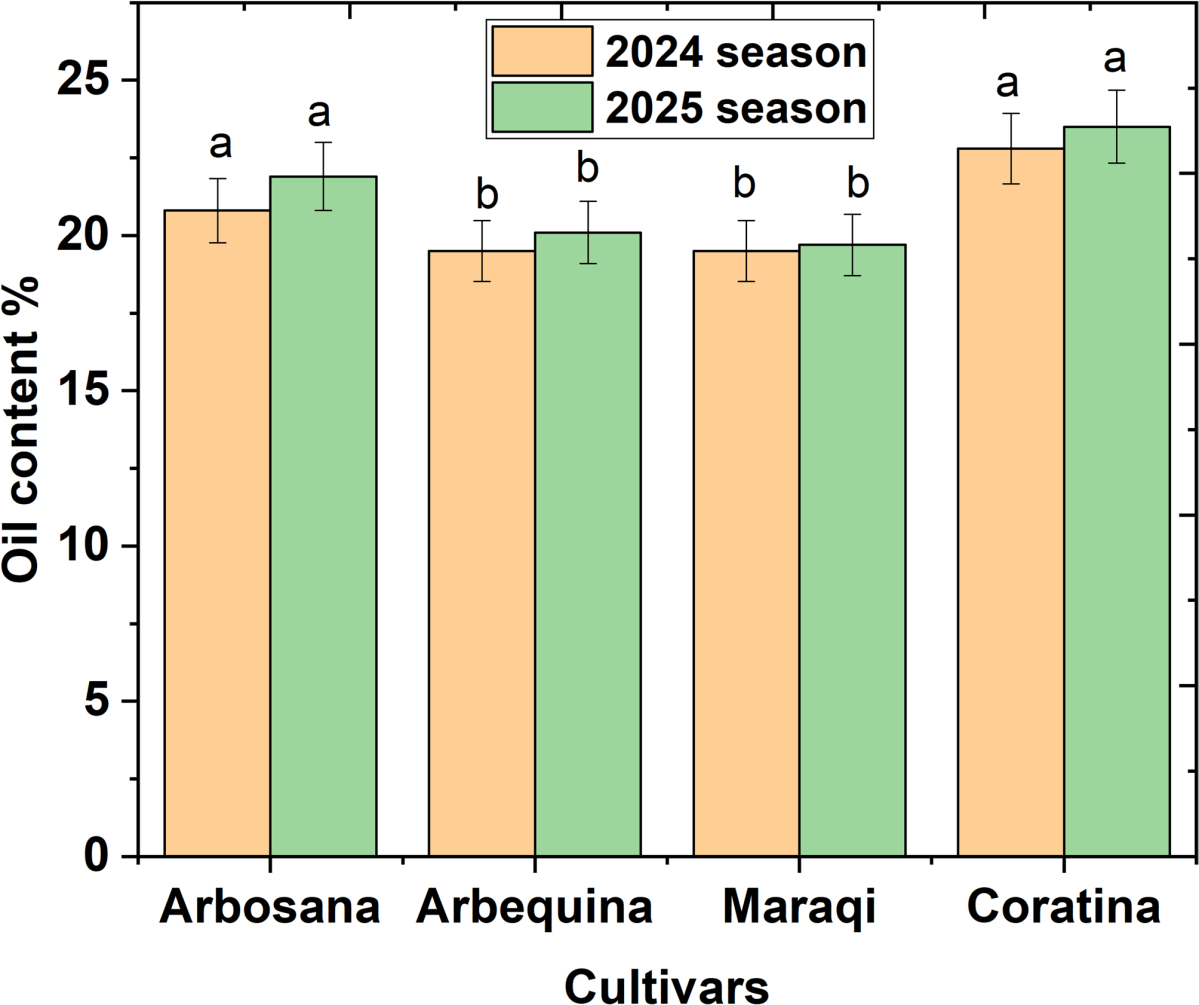
Fruit oil content (% dry weight) of four olive cultivars in the 2024 and 2025 growing seasons. Bars within a season followed by the same letter are not significantly different (*p* ≤ 0.05) according to Duncan’s Multiple Range Test. Two-way ANOVA results: Cultivar effect (F₍₃,₂₄₎ = 112.8, *p* < 0.001); Cultivar × Year interaction (F₍₃,₂₄₎ = 4.56, *p* < 0.05). Error bars represent standard error (SE).

Oil content (% dry weight) showed significant variation among cultivars as shown in (Fig. 6). Coratina’ achieved the highest oil content (52.0% in 2024, 51.0% in 2025), significantly exceeding all other cultivars. ‘Arbequina’ (42.0%, 41.2%) and ‘Maraqi’ (40.0%, 39.5%) registered statistically similar oil contents, forming a middle group. Arbosana showed a tendency toward higher oil content (45.0% in 2024, 46.8% in 2025) and was statistically grouped with Coratina in the second season, though remaining significantly different from Arbequina and Maraqi. The Cultivar × Year interaction was significant (F₍₃,₂₄₎ = 4.56, *p* < 0.05), reflecting ‘Arbosana’s disproportionate increase in oil content from 45.0% in 2024 to 46.8% in 2025 compared to other cultivars.

## Discussion

### Vegetative growth and canopy development

The consistent ranking of cultivars for vegetative growth parameters across both seasons (Coratina > Maraqi > Arbosana/Arbequina) indicates strong genotypic control over growth potential, with minimal Year × Cultivar interaction for these traits (Fig. 1A). Coratina’s superior vegetative expansion (canopy volume 8.2–8.5 m³, shoot length 50.5–52.0 cm) suggests that this cultivar possesses genetic determinants allowing efficient resource capture and biomass accumulation even under the moderate salinity and low fertility conditions of the experimental soil as reported in Sect.  3.1. This aligns with its established reputation as a vigorous and productive cultivar in Mediterranean environments^26^. The observed growth differences reflect genetically determined vigor classes with direct implications for orchard design. Coratina’s vigorous growth necessitates wider spacing (typically ≥ 6 × 6 m) to prevent inter-tree competition and ensure adequate light penetration^26^. In contrast, the compact canopies of Arbequina and Arbosana (3.1–3.6 m³) enable high-density (400–600 trees ha⁻¹) and super-high-density (1500–2500 trees ha⁻¹) planting systems, where reduced vigor is deliberately selected to maximize land-use efficiency and facilitate mechanical harvesting^12,28^. These cultivars’ reduced vegetative effort can be interpreted as a conservative strategy, allocating resources away from excessive growth and potentially toward maintenance or reproduction under stress conditions^28^. Maraqi’s intermediate vigor (5.6–5.8 m³) suggests adaptability to moderate-density systems, though its specific management requirements in intensive plantings remain poorly characterized.

The slight but non-significant increases in all growth parameters from 2024 to 2025 suggest positive orchard establishment and adaptation to site conditions, a common phenomenon in young to mature orchards^16^.

### Physiological strategies for stress adaptation

The concurrent evaluation of chlorophyll content, relative water content (RWC), and proline accumulation allowed differentiation among cultivars’ physiological strategies for coping with the edaphic constraints inherent to newly reclaimed sandy soils (low organic matter, limited water-holding capacity, high evaporative demand, and moderate nutrient limitations). Rather than representing a hierarchical ranking of “stress tolerance,” the observed patterns reflect fundamentally different adaptive strategies, each with distinct physiological costs and benefits.

#### Contrasting adaptive strategies: stress avoidance versus stress tolerance

The results revealed two contrasting physiological profiles among the four cultivars. ‘Arbequina’ and ‘Maraqi’ exhibited a stress-avoidance strategy, characterized by high RWC (86.2–88.5%), minimal proline accumulation (2.8–3.5 µmol g⁻¹ FW), and moderate chlorophyll content. This pattern indicates effective maintenance of tissue hydration through mechanisms such as early stomatal regulation, reduced cuticular transpiration, and possibly superior root architecture that sustains water uptake under soil moisture deficits^27,28^. Stress-avoiding cultivars prioritize the preservation of cellular water status, thereby limiting the need for costly osmotic adjustments. However, this strategy carries an inherent trade-off: sustained stomatal closure, while conserving water, inevitably reduces CO₂ diffusion into leaves, thereby limiting photosynthetic carbon gain and potentially constraining biomass accumulation and yield potential under prolonged stress^29,30^. In contrast, ‘Coratina’ displayed a stress-tolerance strategy, characterized by substantially lower RWC (68.8–75.4%), exceptionally high proline accumulation (12.5–14.2 µmol g⁻¹ FW), and the highest chlorophyll content (SPAD 67.2–68.5). This profile suggests that ‘Coratina’ permits greater tissue dehydration while actively mounting a biochemical tolerance response. The elevated proline serves a dual role: it indicates that the plant has perceived osmotic stress, but more importantly, it represents an active adaptive mechanism functioning as a compatible solute for osmotic adjustment, a reactive oxygen species (ROS) scavenger, and a stabilizer of subcellular structures^10,31^. Thus, ‘Coratina’ invests metabolic energy into osmolyte synthesis to protect cellular machinery under suboptimal hydration, allowing it to sustain photosynthetic activity (evidenced by high SPAD values) and support vigorous vegetative growth and high fruit production even when tissue water status is compromised. The cost of this strategy includes the energetic burden of proline biosynthesis (approximately 30–40 molecules of ATP per molecule of proline synthesized from glutamate) and the potential for long-term metabolic fatigue under repeated or prolonged stress episodes^32,33^. ‘Arbosana’ exhibited intermediate responses (RWC 80.5–82.5%, proline 5.8–6.5 µmol g⁻¹ FW), suggesting a mixed or flexible strategy that may allow moderate stress tolerance without full commitment to either extreme.

#### Isohydric versus anisohydric behavior

The contrasting water-use patterns observed can be interpreted within the well-established framework of isohydric versus anisohydric stomatal regulation. Isohydric plants maintain relatively constant leaf water potential across a range of soil water availabilities by closing stomata early in response to drying soil or increasing vapor pressure deficit^34,35^. This conservative strategy prioritizes the avoidance of low leaf water potential at the expense of carbon gain. The high RWC and low proline levels in ‘Arbequina’ and ‘Maraqi’ are consistent with isohydric behavior: these cultivars appear to close stomata proactively, thereby preserving tissue hydration but potentially limiting photosynthetic carbon assimilation during periods of high evaporative demand^36,37^. Conversely, anisohydric plants allow leaf water potential to decline with soil water deficits, maintaining stomatal openness and photosynthetic activity under conditions that would trigger stomatal closure in isohydric species^34,38^. ‘Coratina’ exhibited low RWC yet sustained high chlorophyll content and vigorous growth, a pattern consistent with anisohydric regulation. By tolerating lower tissue hydration, anisohydric cultivars can maintain carbon gain and support reproductive development under conditions that would induce significant stomatal limitation in isohydric cultivars. However, this comes at the cost of greater cellular dehydration and the associated need for osmotic adjustment through proline accumulation^39,40^. The significant Cultivar × Year interactions observed for RWC and proline (F = 4.87, *p* < 0.01 and F = 5.63, *p* <  0.01, respectively) indicate differential environmental sensitivity, with ‘Coratina’ showing the greatest responsiveness a characteristic typical of anisohydric genotypes that track environmental variation more closely than their isohydric counterparts^35,41^.

#### Carbon allocation trade-offs and yield implications

The physiological strategies identified have direct implications for carbon allocation and reproductive output. ‘Coratina’ invested heavily in both vegetative growth (largest canopy volume, longest shoots) and absolute fruit production (highest yield per tree). This pattern suggests that its anisohydric, stress-tolerant strategy enables continued carbon capture and allocation to sink tissues (shoots, fruits) even under suboptimal hydration. However, the substantial proline accumulation (12.5–14.2 µmol g⁻¹ FW) represents a significant metabolic cost: proline synthesis from glutamate requires NADPH and glutamate, diverting carbon and energy away from growth and reproduction^32^. This cost may partially explain why ‘Coratina’ exhibited the lowest reproductive efficiency (fruit set 0.9–1.1% and yield efficiency 2.6 kg m⁻³) despite its high absolute yield. In contrast, the isohydric, stress-avoiding strategy of ‘Arbequina’ and ‘Arbosana’ prioritized water conservation and tissue hydration over maximum vegetative expansion. Their compact canopies (3.1–3.6 m³) and high yield efficiency (3.6–3.8 kg m⁻³) indicate efficient conversion of limited carbon gain into reproductive structures, with minimal investment in costly osmolyte synthesis. This resource allocation pattern is characteristic of cultivars specifically selected for intensive, high-density planting systems where land-use efficiency and mechanical harvest compatibility are prioritized over individual tree vigor^12,28^.

#### Environmental modulation of strategy expression

It is critical to emphasize that these physiological strategies are not fixed, invariant traits but rather represent the expression of genetic potential modulated by environmental conditions. The significant Cultivar × Year interactions for RWC and proline indicate that the degree of strategy expression varies with inter-annual environmental variation. ‘Coratina’ showed the greatest year-to-year change (4.6% RWC reduction and 13.6% proline increase from 2024 to 2025), suggesting higher phenotypic plasticity and potentially greater sensitivity to environmental fluctuations. ‘Arbequina’ and ‘Maraqi’ demonstrated greater stability across years, which may be advantageous in unpredictable or marginal environments where consistency of performance is valued over maximum potential yield. While previous studies under Mediterranean conditions have classified ‘Coratina’ as a drought-tolerant cultivar based on its performance in deep, well-structured soils^32,38^, our results indicate that under sandy reclaimed soils with limited buffering capacity, this tolerance strategy is accompanied by greater physiological cost, as evidenced by reduced RWC and elevated proline accumulation. This discrepancy highlights the critical role of soil physical properties in modulating cultivar stress responses and underscores the danger of extrapolating cultivar recommendations across dissimilar environments. Performance rankings are not absolute but are contingent upon the specific edaphic and climatic context.

### Reproductive development and yield formation

The reproductive data reveal a classic trade-off between flowering investment and reproductive efficiency as reported in Section “3.3”. Coratina’s prolific flower production (22.5 flowers per inflorescence) represents a high-investment strategy to ensure reproductive success by saturating the environment with potential fruit sites^39^. However, this was accompanied by the highest gynosterility rates (28–30%) and lowest fruit set (0.9–1.1%). This phenomenon is well-documented in some high-flowering-intensity olive cultivars and is often exacerbated by abiotic stresses, which can disrupt floral organ development^40^. The physiological stress experienced by Coratina (low RWC, high proline) may have contributed to the elevated pistil abortion rates, reflecting resource competition between stress responses and reproductive development^41^. Conversely, Arbequina and Arbosana achieved superior fruit set (3.5–4.2%) despite producing fewer flowers (8.5–10.2 per inflorescence), indicating greater reproductive efficiency under the studied conditions. Their stable, low-stress physiological status (high RWC (%), low proline) likely supported more consistent ovule fertility and early fruit development. This aligns with the concept of a “flowering-fruit set trade-off” observed in many fruit tree species, where excessive flower production does not necessarily translate to proportionally higher fruit set due to resource limitations^41^. Maraqi’s intermediate position, coupled with its significant fruit set decline from 2024 to 2025 (21.4%), suggests greater sensitivity to inter-annual environmental variation than the Spanish cultivars. The significant Cultivar × Year interaction for fruit set confirms differential expression of alternate bearing tendencies, with Maraqi showing the least stability.

Yield efficiency (kg fruit m⁻³ canopy) revealed a pattern opposite to absolute yield, with Arbequina (3.8 kg m⁻³) and Arbosana (3.6–3.8 kg m⁻³) demonstrating significantly higher values than Coratina (2.6 kg m⁻³) and Maraqi (2.6–2.8 kg m⁻³) as reported in Section  “3.3.3”. This metric, while valuable for comparing resource-use efficiency, inherently favors cultivars with compact growth habits, as the denominator (canopy volume) is smaller. High yield efficiency in Arbequina and Arbosana indicates dense fruiting wood and effective conversion of vegetative structures into reproductive output characteristics specifically selected for intensive planting systems^42^. However, this should not be interpreted as absolute agronomic superiority, as total orchard productivity depends on the interaction between per-tree efficiency and planting density. In high-density systems, the superior efficiency of Arbequina and Arbosana translates to high per-hectare yields, while in traditional extensive systems; Coratina’s larger individual tree yield may be more advantageous.

### Fruit characteristics and oil content

Fruit size and oil content were strongly genotype-dependent. Coratina’s consistently large fruits (4.4–4.5 g) and high oil content (51–52%) confirm its suitability for oil-focused production systems where tree spacing and resource availability are not limiting^43^. This represents a well-known characteristic of this cultivar, valued for its high pulp-to-pit ratio and oil yield^26^.

Arbosana’s tendency toward higher oil content in 2025 (46.8%), approaching values statistically similar to Coratina, suggests potential advantages under intensive planting systems as reported in Section “3.4.2”. This cultivar’s ability to combine compact growth with relatively high oil content is a primary driver behind its global adoption for super-high-density production systems, where maximum yield efficiency per unit land area, rather than per-tree productivity, is the priority^44^. Arbequina and Maraqi exhibited intermediate oil contents (39.5–42.0%) within commercially acceptable ranges, confirming their reliability for oil production under marginal conditions. It must be emphasized that total oil content, while economically important, represents only one dimension of oil quality. Phenolic composition, which determines oxidative stability and sensory bitterness, varies significantly among cultivars and environments^26,43^. Coratina is renowned for high polyphenol content, while Arbequina typically produces oils with milder sensory profiles. These quality attributes, not evaluated in the present study, should be considered alongside total oil content when matching cultivars to market preferences.

## Conclusion

The results demonstrate that no single cultivar is universally superior; rather, each exhibits distinct strengths and limitations reflecting fundamentally different adaptive strategies: Coratina maximizes absolute productivity (21.2–22.5 kg tree⁻¹, 51–52% oil) but employs a stress-tolerance strategy characterized by active osmotic adjustment through proline accumulation (12.5–14.2 µmol g⁻¹ FW). Rather than indicating stress-induced damage, this proline accumulation represents an adaptive metabolic investment that protects cellular structures and sustains photosynthetic activity despite reduced tissue hydration (RWC 68.8–75.4%). This strategy carries physiological costs, including lower reproductive efficiency (fruit set 0.9–1.1%) and yield efficiency (2.6 kg m⁻³). Arbequina and Arbosana employ a contrasting stress-avoidance strategy, maintaining high RWC (78.9–87.2%) with minimal proline accumulation (3.1–6.5 µmol g⁻¹ FW), thereby conserving metabolic resources that would otherwise be allocated to osmolyte synthesis. This strategy supports superior yield efficiency (3.6–3.8 kg m⁻³) but limits vegetative expansion. Maraqi prioritizes physiological resilience (RWC 86.2–88.5%, proline 2.8-3.0 µmol g⁻¹ FW) over maximum production, though declining fruit set indicates limitations under inter-annual environmental variation. Future research should extend these comparisons under controlled water and edaphic stresses inherent to newly reclaimed sandy soils low organic matter, limited water-holding capacity, moderate nutrient availability^31,32^ and incorporate comprehensive oil quality characterization (phenolic profiles, fatty acid composition, sensory analysis)^26,43^ to further refine cultivar recommendations for marginal environments. Long-term studies are also needed to evaluate cumulative stress effects, yield stability across multiple seasons, and orchard longevity critical considerations for sustainable olive production in newly reclaimed areas.

## Data Availability

The data generated and/or analysed during the current study are available per request to the corresponding author.

## References

[1] Fraga, H., Moriondo, M., Leolini, L. & Santos, J. A. Mediterranean olive orchards under climate change: A review of future impacts and adaptation strategies. *Agronomy* **11**, 56. 10.3390/agronomy11010056 (2020).

[2] Kavvadias, V. & Koubouris, G. Sustainable soil management practices in olive groves. In *Soil Fertility Management for Sustainable Development* 167–188 (Springer, 2019). 10.1007/978-981-13-5904-0_8

[3] Brito, C., Dinis, L. T., Moutinho-Pereira, J. & Correia, C. M. Drought stress effects and olive tree acclimation under a changing climate. *Plants* **8**, 232. 10.3390/plants8070232 (2019). 31319621 10.3390/plants8070232 PMC6681365

[4] Alowaiesh, F., Gad, B. M., Saleh, M., Ali, M. & M. & Integrated use of organic and bio-fertilizers to improve yield and fruit quality of olives grown in low fertility sandy soil in an arid environment. *Phyton* **92**, 1813–1829. 10.32604/phyton.2023.027891 (2023).

[5] Busso, M. A. Biochemical and physiological mechanisms allowing olive trees to survive and produce under water stress conditions. *Lilloa* **60**, 171–188. 10.30550/j.lil/1711 (2023).

[6] Parri, S. Drought and the olive tree in a changing climate: A multi-level response characterisation to explore and valorise Italian cultivars. PhD Thesis. 10.25434/SARA-PARRI_PHD2024-07-26 (2024).

[7] Lo Bianco, R., Proietti, P., Regni, L. & Caruso, T. Planting systems for modern olive growing: Strengths and weaknesses. *Agriculture* **11**, 494. 10.3390/agriculture11060494 (2021).

[8] Guermazi, E., Wali, A. & Ksibi, M. Combining remote sensing, SPAD readings, and laboratory analysis for monitoring olive groves and olive oil quality. *Precision Agric.* **25**, 65–82. 10.1007/s11119-023-10058-6 (2024).

[9] Karimi, S., Rahemi, M., Rostami, A. A. & Sedaghat, S. Drought effects on growth, water content and osmoprotectants in four olive cultivars with different drought tolerance. *Int. J. Fruit Sci.* **18**, 254–267. 10.1080/15538362.2018.1438328 (2018).

[10] Spormann, S., Nadais, P., Sousa, F., Pinto, M., Martins, M., Sousa, B. & Soares, C. Accumulation of proline in plants under contaminated soils—are we on the same page? *Antioxidants* **12**, 666 (2023). 10.3390/antiox12030666. 10.3390/antiox12030666 PMC10045403 36978914

[11] Gucci, R. et al. Fruit growth, yield and oil quality changes induced by deficit irrigation at different stages of olive fruit development. *Agric. Water Manag.* **212**, 88–98. 10.1016/j.agwat.2018.08.022 (2019).

[12] Camposeo, S., Vivaldi, G. A., Montemurro, C., Fanelli, V. & Cunill Canal, M. Lecciana, a new low-vigour olive cultivar suitable for super high density orchards and for nutraceutical EVOO production. *Agronomy* **11**, 2154. 10.3390/agronomy11112154 (2021).

[13] Molinu, M. G., Deiana, P., Dettori, S., Mercenaro, L., Nieddu, G., Dore, A. & Santona, M. Looking for typical traits in monovarietal VOOs according to their phenolic composition. *Foods* **13**, 3425 (2024). 10.3390/foods13213425. 10.3390/foods13213425 PMC11545162 39517209

[14] Elsorady, M. E., Mohamed, A. S. & Mohamed, E. S. Influence of organic fertilization and irrigation on quality of maraqi cultivar virgin olive oil. *Egypt. J. Agricult. Res.* **92**, 709–728. 10.21608/ejar.2014.155874 (2014).

[15] Peel, M. C., Finlayson, B. L. & McMahon, T. A. Updated world map of the Köppen–Geiger climate classification. *Hydrol. Earth Syst. Sci.* **11**, 1633–1644. 10.5194/hess-11-1633-2007 (2007).

[16] Fernández-Escobar, R. Olive nutritional status and tolerance to biotic and abiotic stresses. *Front. Plant Sci.* **10**, 1151. 10.3389/fpls.2019.01151 (2019). 31608093 10.3389/fpls.2019.01151 PMC6769400

[17] Rosati, A., Wolz, K. J., Murphy, L., Ponti, L. & Jose, S. Modeling light below tree canopies overestimates net photosynthesis and radiation use efficiency in understory crops by averaging light in space and time. *Agric. For. Meteorol.* **284**, 107892. 10.1016/j.agrformet.2019.107892 (2020).

[18] Křížová, K. et al. Using a single-board computer as a low-cost instrument for SPAD value estimation through colour images and chlorophyll-related spectral indices. *Ecol. Inf.* **67**, 101496. 10.1016/j.ecoinf.2021.101496 (2022).

[19] Slama, A., Mallek-Maalej, E., Ben Mohamed, H., Rhim, T. & Radhouane, L. A return to the genetic heritage of durum wheat to cope with drought heightened by climate change. *PLoS One*. **13**, e0196873. 10.1371/journal.pone.0196873 (2018). 29795584 10.1371/journal.pone.0196873 PMC5967785

[20] Bates, L. S., Waldren, R. P. A. & Teare, I. D. Rapid determination of free proline for water-stress studies. *Plant. Soil.* **39**, 205–207. 10.1007/BF00018060 (1973).

[21] Seifi, E., Guerin, J., Kaiser, B. & Sedgley, M. Flowering and fruit set in olive: A review. *Iran. J. Plant. Physiol.* **5**, 1263–1272 (2015).

[22] Montemurro, C., Dambruoso, G., Bottalico, G. & Sabetta, W. Self-incompatibility assessment of some Italian olive genotypes (*Olea europaea* L.) and cross-derived seedling selection by SSR markers on seed endosperms. *Front. Plant Sci.* **10**, 451. 10.3389/fpls.2019.00451 (2019). 31031787 10.3389/fpls.2019.00451 PMC6473062

[23] Vidal, A. M., Moya, M., Alcalá, S., Romero, I. & Espínola, F. Enrichment of refined olive oils with phenolic extracts of olive leaf and exhausted olive pomace. *Antioxidants* **11**, 204. 10.3390/antiox11020204 (2022). 35204087 10.3390/antiox11020204 PMC8868085

[24] Famiani, F. et al. Deflowering as a tool to accelerate growth of young trees in both intensive and super-high-density olive orchards. *Agronomy* **12** (2319). 10.3390/agronomy12102319 (2022).

[25] Feldsine, P., Abeyta, C. & Andrews, W. H. AOAC INTERNATIONAL methods committee guidelines for validation of qualitative and quantitative food microbiological official methods of analysis. *J. AOAC Int.* **85**, 1187–1200. 10.1093/jaoac/85.5.1187 (2002). 12374420

[26] Clodoveo, M. L. et al. Research and innovative approaches to obtain virgin olive oils with a higher level of bioactive constituents. *Olive Olive Oil Bioact. Const.* 179–215 (Elsevier, 2015). 10.1016/B978-1-63067-041-2.50013-6.

[27] Flexas, J. et al. Stomatal and mesophyll conductances to CO_2_ in different plant groups: underrated factors for predicting leaf photosynthesis responses to climate change? *Plant. Sci.* **226**, 41–48. 10.1016/j.plantsci.2014.06.011 (2014). 25113449 10.1016/j.plantsci.2014.06.011

[28] Morcillo Juliá, L. et al. Post-drought conditions and hydraulic dysfunction determine tree resilience and mortality across Mediterranean Aleppo pine (Pinus halepensis) populations after an extreme drought event. *Tree Physiol.* **42** (8), 1573–1588. 10.1093/treephys/tpac029 (2022). 10.1093/treephys/tpac001 35038335

[29] Medrano, H. et al. From leaf to whole-plant water use efficiency (WUE) in complex canopies: Limitations of leaf WUE as a selection target. *Crop J.* **3** (3), 220–228. 10.1016/j.cj.2015.04.003 (2015).

[30] Chaves, M. M., Flexas, J. & Pinheiro, C. Photosynthesis under drought and salt stress: Regulation mechanisms from whole plant to cell. *Ann. Bot.* **103** (4), 551–560. 10.1093/aob/mcn125 (2009). 18662937 10.1093/aob/mcn125 PMC2707345

[31] Hayat, S. et al. Role of proline under changing environments: A review. *Plant. Signal. Behav.* **7** (11), 1456–1466. 10.4161/psb.21949 (2012). 22951402 10.4161/psb.21949 PMC3548871

[32] Verslues, P. E. & Sharma, S. Proline metabolism and its implications for plant-environment interaction. *Arabidopsis Book.* **8**, e0140. 10.1199/tab.0140 (2010). 22303265 10.1199/tab.0140 PMC3244962

[33] Pallag, G. et al. Proline oxidation supports mitochondrial ATP production when complex I is inhibited. *Int. J. Mol. Sci.* **23** (9), 5111. 10.3390/ijms23095111 (2022). 35563503 10.3390/ijms23095111 PMC9106064

[34] Tardieu, F., Simonneau, T. & Muller, B. The physiological basis of drought tolerance in crop plants: A scenario-dependent probabilistic approach. *Annu. Rev. Plant. Biol.* **69**, 733–759. 10.1146/annurev-arplant-042817-040218 (2018). 29553801 10.1146/annurev-arplant-042817-040218

[35] Martinez-Vilalta, J. & Garcia-Forner, N. Water potential regulation, stomatal behaviour and hydraulic transport under drought: Deconstructing the iso/anisohydric concept. *Plant. Cell. Environ.* **40** (6), 962–976. 10.1111/pce.12846 (2017). 27739594 10.1111/pce.12846

[36] Gholami, R., Fahadi Hoveizeh, N., Zahedi, S. M., Gholami, H. & Carillo, P. Effect of three water-regimes on morpho-physiological, biochemical and yield responses of local and foreign olive cultivars under field conditions. *BMC Plant. Biol.* **22**, 477. 10.1186/s12870-022-03865-2 (2022). 36203130 10.1186/s12870-022-03855-8 PMC9540738

[37] Razouk, R., Hssaini, L., Alghoum, M., Adiba, A. & Hamdani, A. Phenotyping olive cultivars for drought tolerance using leaf macro-characteristics. *Horticulturae* **8**, 939. 10.3390/horticulturae8100939 (2022).

[38] Sade, N., Gebremedhin, A. & Moshelion, M. Risk-taking plants: Anisohydric behavior as a stress-resistance trait. *Plant. Signal. Behav.* **7** (7), 767–770. 10.4161/psb.20505 (2012). 22751307 10.4161/psb.20505 PMC3583960

[39] Poury, N., Seifi, E. & Alizadeh, M. Effects of salinity and proline on growth and physiological characteristics of three olive cultivars. *Gesunde Pflanzen*. **75**, 1169–1180. 10.1007/s10343-022-00767-3 (2023).

[40] Ben Abdallah, M., Methenni, K., Taamalli, W. & Ben Youssef, N. Post-stress recovery from drought and salinity in olive plants is an active process associated to physiological and metabolic changes. *Acta Physiol. Plant.* **46**, 120. 10.1007/s11738-024-03743-2 (2024).

[41] Hochberg, U., Rockwell, F. E., Holbrook, N. M. & Cochard, H. Iso/anisohydry: A plant–environment interaction rather than a simple hydraulic trait. *Trends Plant. Sci.* **23** (2), 112–120. 10.1016/j.tplants.2017.11.002 (2018). 29223922 10.1016/j.tplants.2017.11.002

[42] Trentacoste, E. R. et al. Effect of regulated deficit irrigation during the vegetative growth period on shoot elongation and oil yield components in olive hedgerows (cv. Arbosana) pruned annually on alternate sides in San Juan, Argentina. *Irrig. Sci.* **37**, 533–546. 10.1007/s00271-019-00639-5 (2019).

[43] Cecchi, L. et al. Virgin olive oil by-product valorization: An insight into the phenolic composition of olive seed extracts from three cultivars as sources of bioactive. *Molecules* **28**, 2776. 10.3390/molecules28062776 (2023). 36985747 10.3390/molecules28062776 PMC10059698

[44] Tous, J. Wiley,. The influence of growing region and cultivar on olives and olive oil characteristics and on their functional constituents. In *Olives and Olive Oil as Functional Foods* (eds Shahidi, F. & Kiritsakis, A.) 45–80 (2017). 10.1002/9781119135340.ch4

